# Supplementary material for: Efficient Reduction Photocatalyst of 4-Nitrophenol Based on Ag-Nanoparticles-Doped Porous ZnO Heterostructure
Source: Nanomaterials (Basel). 2022 Aug 19;12(16):2863. doi: 10.3390/nano12162863 (PMC9415390; doi:10.3390/nano12162863)
Supplement: Supplementary file 1 [file nanomaterials-12-02863-s001.zip › nanomaterials-1856125-supplementary.pdf]

nanomaterials

Article

Efficient Reduction Photocatalyst of 4-Nitrophenol Based on Ag-Nanoparticles-Doped Porous ZnO Heterostructure

Shali Lin, Xiaohu Mi, Lei Xi, Jinping Li, Lei Yan, Zhengkun Fu * and Hairong Zheng

School of Physics and Information Technology, Shaanxi Normal University, Xi'an 710119, China
* Correspondence: zkfu@snnu.edu.cn

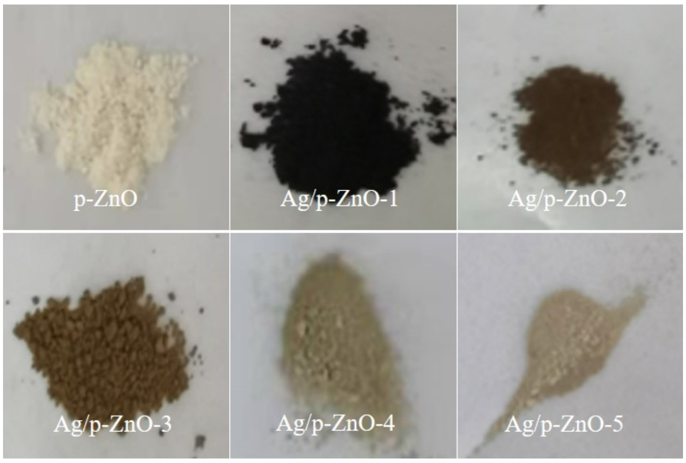

Figure S1. Digital photos of p-ZnO with different Ag NPs loading.

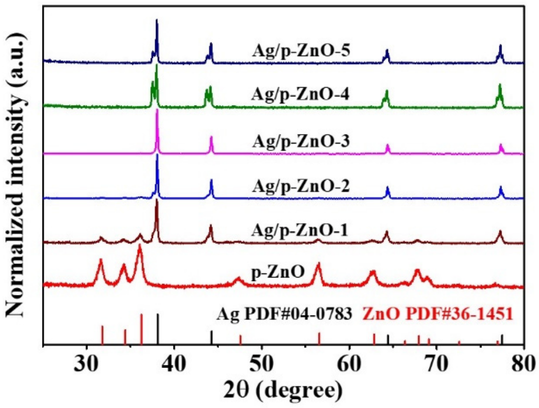

Figure S2. XRD patterns of Ag/p-ZnO with different Ag NPs loading.

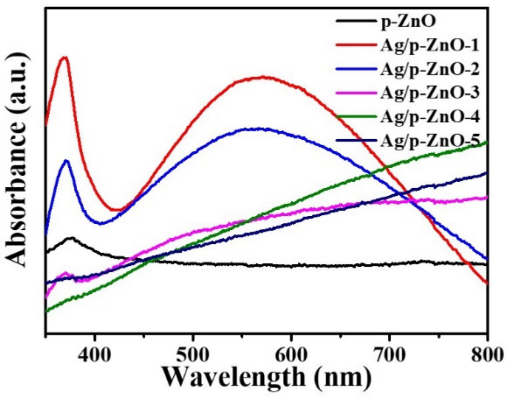

Figure S3. This is a figure. Schemes follow the same formatting. The UV-visible absorption spectra of Ag/p-ZnO with different Ag NPs loading.

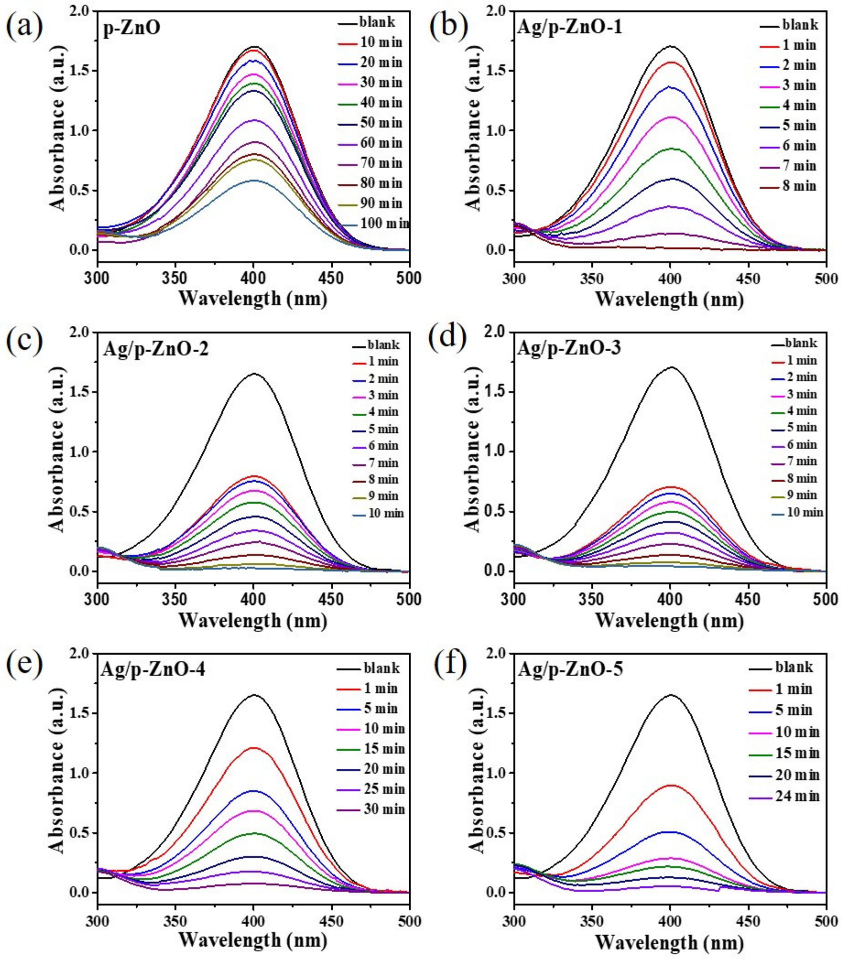

Figure S4. Time-dependent UV-vis spectra showing gradual reduction of 4-NP over Ag/p-ZnO collected at 1 min intervals continuously under natural light.

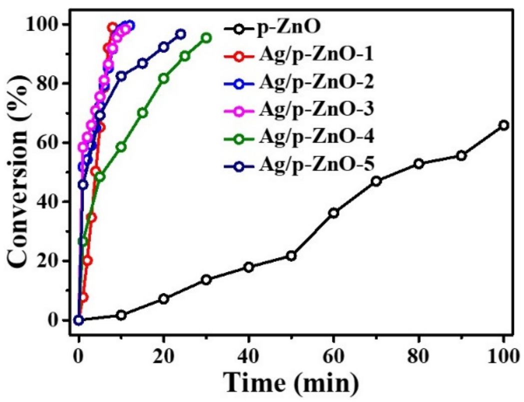

Figure S5. Catalytic conversion of 4-NP over Ag/p-ZnO heterostructure under natural light.

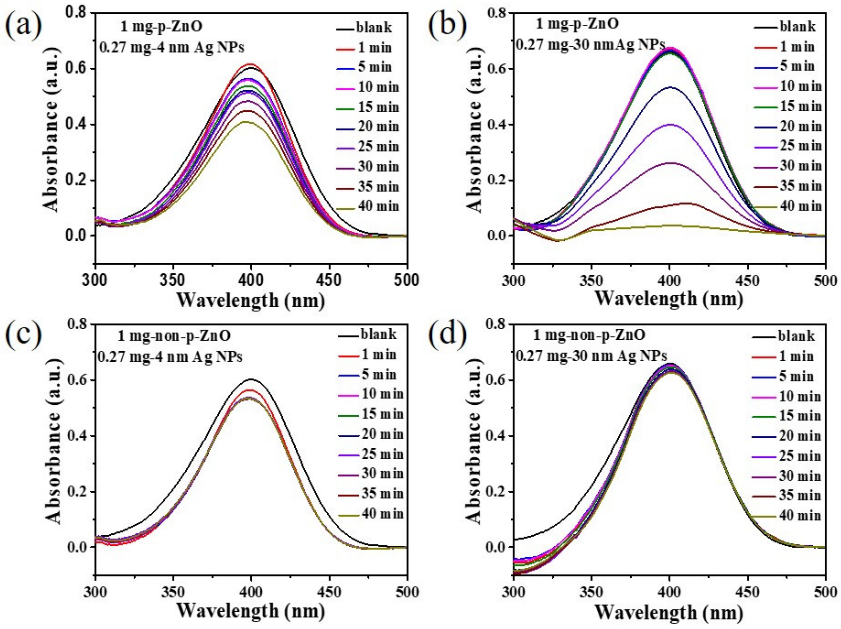

Figure S6. Comparative mixture catalysts collected at 1 min intervals continuously under natural light. (a) 1 mg-p-ZnO + 0.27 mg 4 nm Ag NPs, (b) 1 mg-p-ZnO + 0.27 mg 30 nm Ag NPs, (c) 1 mg-non-p-ZnO + 0.27 mg 4 nm Ag NPs, (d)1 mg-non-p-ZnO + 0.27 mg 30 nm Ag NPs.

Table S1. Sample mass and concentration of reaction solution required for catalytic reaction.

Catalyst / Solution	Ag/p-ZnO	p-ZnO 4 nm Ag NPs	p-ZnO 30 nm Ag NPs	non-p-ZnO 4 nm Ag NPs	non-p-ZnO 30 nm Ag NPs	p-ZnO
Ag/p-ZnO	1 mg	0	0	0	0	0
p-ZnO	0	1 mg	1 mg	0	0	1 mg
non-p-ZnO	0	0	0	1 mg	1 mg	0
4 nm Ag NPs (0.27 mg/mL)	0	1 mL	0	1 mL	0	0
30 nm Ag NPs (0.27 mg/mL) mg/mL)	0	0	1 mL	0	1 mL	0
4-NP (0.4 mM/mL)	1 mL	1 mL	1 mL	1 mL	1 mL	1 mL
$NaBH_4$ (40 mM/mL)	1 mL	1 mL	1 mL	1 mL	1 mL	1 mL
DI-water/mL	0	2.705 mL	2.705 mL	2.705 mL	2.705 mL	2.705 mL
Toal volume/ mL	2 mL	5.705 mL	5.705 mL	5.705 mL	5.705 mL	5.705 mL

Table S2. The rate constants kapp (min-1) and conversion rate calculated from Fig.5 and Fig.S5.

Sample	k_{app} (min^{-1})	Conversion (%)
p-ZnO	0.011 ± 0.001	65.9 ± 0.0
Ag/ZnO-1	0.482 ± 0.095	99.0 ± 0.2
Ag/ZnO-2	0.432 ± 0.041	99.6 ± 0.0
Ag/ZnO-3	0.334 ± 0.027	98.4 ± 0.3
Ag/ZnO-4	0.091 ± 0.007	95.4 ± 0.0
Ag/ZnO-5	0.122 ± 0.011	96.8 ± 0.2
